# Bean Preferences Vary by Acculturation Level among Latinas and by Ethnicity with Non-Hispanic White Women

**DOI:** 10.3390/ijerph17062100

**Published:** 2020-03-22

**Authors:** Michelle M. Heer, Donna M. Winham

**Affiliations:** Food Science & Human Nutrition, Iowa State University, Ames, IA 50010, USA; mmheer@gmail.com

**Keywords:** pulses, food security, canned foods, Mexican Americans, dietary acculturation, consumer preferences, immigrants, health disparities, nutrition knowledge

## Abstract

With high levels of protein, fiber, folate, iron and other micronutrients, the Dietary Guidelines for Americans recommends eating beans for optimal nutrition. Low-income women are at greater risk of nutrition-related health disparities. Use of beans may change among Hispanic women (Latinas) during acculturation, but few studies exist that describe specific preferences of this important traditional food. Preserving or promoting beans in the diets of all low-income women could improve dietary quality. The study objectives were to describe consumption frequency, purchasing patterns, and attitudes toward dry and canned beans, by acculturation level among Latinas and by ethnicity with non-Hispanic White women. Survey data were collected from 356 women (µ 32 y ± 9 y; 81% Latina), who were enrolled in, or eligible for, a federal nutrition assistance, or unemployment, program in Phoenix, Arizona, USA. Participants had positive attitudes toward beans overall. Less acculturated and bicultural Latinas bought dry beans more often than their peers. Price was considered important in canned bean selection for Non-Hispanic White women, and less acculturated Latinas had poorer attitudes toward canned. Awareness of these attitudes and preferred traits of low-income women suggests ways to message populations to maintain or increase bean consumption. Negative views of canned beans by Latinas should be investigated further. Inclusion of canned beans in nutrition assistance programs may benefit those unfamiliar with preparing dry beans.

## 1. Introduction

Beans have been a central element of Mexican and Central American cuisine since pre-Hispanic times [1], and annual per-capita dry-bean consumption is nearly four times greater in Mexico (11 kg) than in the United States (US) (3 kg) [2]. When people from Latin American countries immigrate to the US, their consumption of beans and other traditional foods may decline over time with acculturation and adaptation to new socio-ecological environments [3,4,5]. Due to variability in methodology, Latino subgrouping, and geographic location, dietary acculturation research has yielded conflicting results, although some constant dietary changes have been identified [3]. Consistent alterations with time include increased intake of sugar-sweetened beverages and fast foods and decreased consumption of fruits, rice, and beans [3], all of which can elevate chronic disease risk, especially when coupled with poverty and race-related health inequities associated with Latinos in the US [6,7,8]. The US government demographic classification ‘Hispanic’ refers to people with origins in a Spanish-speaking country regardless of personal cultural affiliation or language [9]. In this research, ‘Latinas’ describes women who retain some degree of Latin American cultural practices and ethnic identity. By definition, the termHispanic is used in the context of government statistics and describes ancestral origin, not necessarily cultural affiliation [9].

Inclusion of beans in the diets of all population groups, including Hispanic immigrants, is a positive approach which can improve dietary quality, increase vegetable intake and lower chronic-disease risk [5,7]. Beans are noted for their high fiber, protein, folate, iron, potassium, and magnesium content [7,10,11]. Except for protein, these essential nutrients are lacking in the diets of many Americans [10,11]. National survey data show that, on any given day, less than 8% of all US adults, compared to 25% of Hispanic Americans, consume pulses [7,10]. Pulses, defined as the edible seeds from mature pods, include dry beans, peas, lentils, chickpeas, and black-eyed peas. “Dry beans” refers to bean seeds both dried and bagged, and those cooked and canned, but does not include green or string beans eaten as pods [7,11]. Popular dry bean varieties in the US are pinto, black, navy, white, and kidney [11]. Pulse production also contributes to sustainable agriculture and improved nutrition globally [12].

The 2015 Dietary Guidelines for Americans (DGA) mention consuming pulses for dietary quality and better health [10]. The Special Supplemental Food Program for Women, Infants and Children (WIC) includes them in their food packages, [13,14] and the Expanded Food and Nutrition Education Program (EFNEP) curriculum features pulses in its national lesson plan as alternative protein choices [14]. These programs focus on reducing disparities in food security and improving nutrition knowledge, primarily for low-income and at-risk women with children [14]. Promoting canned beans as well as dry is a way to reduce meal preparation time and increase access to these healthy foods [13,15].

Important factors in reducing health disparities among Latinas may include identifying preferences for bean purchases (type, form, product size, origin), attitudes toward bean consumption, and degree of acculturation [5,15]. Understanding the preferences and attitudes of all low-income women can provide additional insight into overall family consumption patterns [16,17]. While private companies may have marketing research data, this information is not publicly available and may represent views of consumers who are not low-income. Few studies have investigated the attitudes or purchasing preferences of low-income women, specifically Latinas, regarding beans or the other foods obtained through food-assistance programs [15,16,18,19]. This paucity of detail interferes with the capability of nutritionists and health professionals to effectively promote pulse consumption [15,20]. Nutrition education programs that account for women’s established opinions can leverage beneficial behaviors and dispel myths and misconceptions through tailored messages [21]. Using cultural rituals in nutrition programming can also increase the appeal of traditional foods [22].

While about 16% of the US population in 2010 was Hispanic, they comprised about 30% of Arizona residents [23]. In Maricopa county, roughly 66% of female Arizona WIC clients and 52% of Maricopa county EFNEP participants self-reported as Hispanic in 2011, when these data were collected [24,25]. To address research gaps on factors that influence bean consumption, such as acculturation level, ethnicity, preferred dry and canned bean purchase characteristics, knowledge and attitudes, and to help guide service improvements of related Arizona agencies, e.g., EFNEP, a survey was administered to low-income Hispanic and non-Hispanic White (NHW) women in metropolitan Phoenix, Arizona. The survey’s objectives were to: 1) compare consumption frequencies; 2) assess purchasing preferences; and 3) describe attitudes toward beans, in general and to canned beans, in terms of acculturation levels and ethnicity among low-income Latinas and non-Hispanic White women. It was hypothesized that dry and canned bean consumption, purchasing preferences, and attitude scores would vary by acculturation status and ethnicity.

## 2. Materials and Methods 

### 2.1. Participants and Survey Development

Women aged 18–55 years who were currently enrolled or eligible to participate in a metropolitan Phoenix federal assistance program (WIC, EFNEP, Temporary Aid for Needy Families, or SNAP), were recruited between May and December 2011. Data collection was conducted at EFNEP classes, at a WIC clinic with a high percentage of Hispanic clientele and an unemployment job center. 

The survey was developed based on a literature review, previous research [26], two focus groups with EFNEP participants (*n* = 24), and one focus group with extension instructional specialists (*n* = 7) who were laypersons from the communities served. Focus groups were moderated by the co-author (D.M.W.) and a bilingual/bicultural EFNEP research staff member. Two EFNEP participants preferred to respond in Spanish, but had some English fluency. Audio recordings were transcribed by the collaborating EFNEP researcher. Topics discussed were views of beans, cooking practices, role of beans in traditional diets, preference for certain bean characteristics based on culture, cultural acceptability, and perceived barriers and motivators to bean consumption. Seven Likert-type general-bean questions related to perceptions about taste, association of beans with poor people, if friends eat beans, issues with gas, and ease of preparation of dry beans, were asked. A second set of seven Likert-type questions related to taste, cultural and family acceptance, nutrition, and cost of canned beans were also asked. Response categories for these statements were: strongly disagree, disagree, neutral, agree, and strongly agree. 

Eight public health nutrition researchers and three EFNEP field staff evaluated the semi-final survey instrument for content validity. All bean-related questions were translated by a bilingual/bicultural staff member, then back-translated by an external consultant. A pilot test with three different EFNEP classes (*n* = 23) allowed participants to provide feedback and discuss points of confusion or meaning on the questions in general, and the Spanish wording. The co-author and the EFNEP bilingual researcher led these sessions also. Pilot test participant data were excluded from analysis. Before official data collection began, minor format and word changes were made based on pilot input. Survey findings regarding the knowledge of bean-related health benefits, utilization of food assistance programs, and health behaviors have been previously reported [27,28,29].

Acculturation status was evaluated by the Bidimensional Acculturation Scale (BAS) [30]. Latinas were classified into three groups: Hispanic-dominant (less acculturated), bicultural, and English-dominant (more acculturated) [30]. Non-Hispanic White women (NHW) were their own category. For questions on demographics, ethnicity, race, household characteristics, and food costs, the standard EFNEP enrollment form was used [31]. Respondents completed the USDA Core Food Security Module to measure food security [32,33]. The validated English and Spanish versions of these two instruments were used. Participants were read a verbal script in English or Spanish prior to receiving the survey whose completion was considered informed consent. No personal identification information was obtained from participants. The Arizona State University Institutional Review Board deemed the study exempt. 

### 2.2. Survey Administration and Data Collection 

Self-administration of the surveys occurred at the end of the third or fourth session of the standard EFNEP six-class series before beans and other pulses were specifically discussed. Bilingual EFNEP instructional specialists were available to aid participants with form completion. Small incentives, e.g., grocery coupons, writing pads, pens, totes, etc., were given at survey completion. Two to four staff personnel, including at least one bilingual staff person, set up a desk, placed information signs, and distributed information to waiting clients at the WIC clinic and the unemployment center. Individuals completing the survey, at either the WIC clinic or the unemployment center, received a $3.00 cash incentive. 

### 2.3. Data Transformations and Statistical Analysis 

Principal components analysis with varimax rotation of the 14 Likert statements produced four factors with eigenvalues greater than 1.0 and explained 59.3% of the variance. Factor loadings less than 0.40 were suppressed [34]. Factor 1 was general bean attitudes (eigenvalue 3.89, 27.82% variance). Factor 2 and Factor 3 (eigenvalues 2.15, 1.25; 15.35%, 8.93% variance, respectively) were both canned-bean related variables. Only two items loaded on Factor 4 (eigenvalue 1.004; 7.17% variance). Reliability analysis resulted in a five-item general-bean scale (Cronbach’s alpha = 0.78) from Factor 1, and a six-item canned-bean scale (Cronbach’s alpha = 0.76) as a combination of items in Factor 2 and 3 [34]. The Likert-type responses were analyzed in their original five categories using Chi-square and ANOVA by ethnicity and acculturation level. For clarity of display, responses were condensed into 3-categories of ‘disagree, neutral, agree’ in the results section. A general linear model representing acculturation level, age, education, household size, food security score and bean-consumption frequency was included in predictive models of general-bean and canned-bean attitude scale scores. Data analysis utilized SPSS V. 25 (IBM, Armonk, NY, USA) software, with a significance level of *p* < 0.050. 

## 3. Results

The completion rate for eligible women was 90% (356/397) with 44% of the total sample recruited from WIC, 42% from EFNEP, and 14% from the job center. The majority of the 356 participants self-identified as Hispanic (81%), with 91% of these stating Mexican or Mexican American ancestry, and 66% choosing to complete the survey in Spanish. Ninety-seven percent of the less acculturated and 62% of the bicultural Latinas were born outside the US, whereas 95% of the more acculturated Latinas and 98.7% of NHW women were US-born (*p* < 0.001). Less acculturated Latinas had fewer years of education, larger household size, more children, and a higher percentage were married in comparison to their peers (all *p* < 0.001; Table 1). More acculturated Latinas were significantly younger (*p* < 0.001), and more likely to be unmarried than their peers (*p* < 0.001). The less acculturated Latinas had the highest percentage in the ‘low’ food security category, but the more acculturated Latinas had the largest percentage reporting ‘very low’ food security (*p* = 0.008). 

Higher percentages of the less acculturated and bicultural Latinas purchased beans of any type, consumed them three or more times per week, and had knowledge of dry bean preparation than the more acculturated Latinas and NHW women (*p* < 0.001 for all). All of the Latinas were more likely to have someone in their household cook dry beans at least 1–2 times per week (*p* < 0.001) (Table 2).

Dry and canned purchase patterns were significantly different by acculturation and ethnicity. More of the less acculturated Latinas bought dry beans than any other group (*p* < 0.001). Of the women who purchased dry beans, less acculturated Latinas preferred them loose or in bulk, whereas NHW women bought beans in small 1–2 pound bags (*p* < 0.001 for both). The selection characteristic of tradition-based purchase of dry beans (*p* < 0.001) was more important to less acculturated and more acculturated Latinas than for the bicultural or NHW women. Quick-cooking beans were preferred by less acculturated Latinas and NHW women in contrast to bicultural and more acculturated Latinas (*p* = 0.037). A greater number of less acculturated and bicultural Latinas preferred beans grown in Mexico or Central America than other women (*p* < 0.001).

Of those who reported buying canned beans, less acculturated Latinas purchased them significantly less often when compared to the other three groups (*p* < 0.001). Price was an important factor in canned-bean selection for the NHW women but not as much for all of the Latinas (*p* < 0.001). Twenty-six percent of all women said they did not buy a particular brand, and 13% stated they did not remember the brand name. About 19% purchased Rosarita (Conagra, Chicago, IL, USA), 10% La Costeña (Tucson, AZ, USA), 6.5% Sun Vista (Faribault, MN, USA), and 6% Goya (Secaucus, NJ, USA) brands. Twenty percent mentioned a wide range of other various brands (data not shown). 

Regardless of canned or dry form, women were asked how often they consumed ten nationally popular market classes of pulses [2,7]. Pinto beans were most frequently consumed at least twice per month (78.3%), with lentils a distant second (32.8%). Black (25.1%), baked (23.9%), mayocoba (22.5%), chickpeas (19.5%), navy (13.3%), red kidney (11.7%), black eyed peas (7.1%), and fava beans (6.4%) were eaten by fewer women. Pinto, lentil, and mayocoba intakes were significantly higher for the less acculturated and bicultural Latinas in comparison to more acculturated Latinas and NHWs (*p* < 0.001). Chickpea consumption (*p =* 0.012) was significantly lower for the more acculturated Latinas than the other women. Red kidney beans were eaten most frequently by NHWs and rarely by less acculturated Latinas (*p* < 0.001). NHWs and more acculturated Latinas reported higher baked bean consumption than bicultural and less acculturated Latinas (*p =* 0.001), whereas fava bean intakes were mainly among less acculturated Latinas (*p =* 0.005). There were no observed acculturation or ethnic differences for black, navy, or black eyed pea intakes. 

The general-bean and canned-bean attitude responses for Likert-type statements are displayed in Table 3. Significant acculturation level/ethnicity related differences were found for 12 of the 14 statements. A greater percentage of Latinas compared to NHW women disagreed that it is difficult to make food with beans (*p* = 0.005), and that they take too long to prepare (*p* < 0.001). While about half of all the women agreed beans can cause intestinal gas, a higher percentage of the less acculturated Latinas than NHW women disagreed with this statement (*p* < 0.01). A larger number of the bicultural and more acculturated Latinas disagreed with the assertion that their family/children refuse to eat beans (*p* < 0.01), and that beans are not eaten by their friends (*p* = 0.013) compared to the less acculturated and the NHW women. When asked about reasons that might prevent them from eating or cooking with canned beans, less acculturated Latinas were more likely to agree that canned beans are not true to their culture, their families will not eat them, they would go without beans if not cooking them themselves, and that canned beans are not healthy, are too expensive, do not taste good, and contain preservatives when compared to the responses from all other groups (*p* < 0.001 for all).

Less acculturated Latinas and NHW women had significantly lower or less positive bean attitude scores compared to bicultural and more acculturated Latinas (*p* < 0.001). A general linear model (GLM) tested whether six variables (age, food security score, education level, number of children in household, acculturation category, and bean consumption frequency) could be used to predict a bean-attitude scale. Results supported that 16.4% of the general bean attitude score variance (R^2^ = 0.164; F (22,318) = 2.84, *p* = 0.000; partial η^2^ = 0.164; power = 1.00) was explained by the model. The general bean attitude scale was predicted by bean consumption frequency (F(4) = 3.73; *p* = 0.006; partial η^2^= 0.045; power = 0.884), and acculturation level (F(3) = 3.006; *p* = 0.031; partial η^2^= 0.028; power = 0.706).

The average canned-bean attitude scale was significantly lower or less positive for less acculturated Latinas than for any other group (*p* < 0.001). The GLM results for the canned-bean attitude scale indicated 24.6% of the observed differences in the canned bean-attitude scale was explained by the model (R^2^ = 0.246; F (22,318) = 4.720, p = 0.000; partial η^2^ = 0.246; power = 1.00). Acculturation level was predictive of canned-bean attitude score (F(3) = 13.89; *p* = 0.000; partial η^2^= 0.116; power = 1.00).

## 4. Discussion

The findings support the hypothesis that dry and canned bean consumption, purchasing preferences, and attitude scores vary by acculturation status and ethnicity among low-income Latinas and NHW women. Differences in variable values corresponded to categorical acculturation levels progressing from less acculturated to bicultural to more acculturated Latinas with respect to consumption frequencies, purchasing preferences, knowledge of preparation, and attitudes towards beans and canned beans. These trends are consistent with other studies showing more acculturated US Latinos, notably Mexican Americans, as well as Puerto Ricans, are eating fewer beans over time [5,14,19]. However, the current study is one of a few with a large enough sample size to characterize the nuances of acculturation and bean consumption patterns.

The most positive general-bean attitude scores were in the bicultural and more acculturated Latinas rather than in the less acculturated group, suggesting beans remain a strong marker of Latino cultural identity independent of reported consumption, as observed in additional studies [5,14]. The positive general-bean attitude scores may be due to exposure of healthy bean messaging in other mainstream media programs not received by less acculturated Latinas. In any case, such positive attitudes among “acculturating” women may serve as a leverage point for achieving greater consumption through appropriate messaging that contributes to the cultural and nutritional value of beans and other pulses [5,19]. As part of the nutrition transition even within Mexico, a recent study found positive attitudes about legumes for their taste, health benefits, and nutrition, despite consumption levels lower than recommendations [35].

Based on previous research, Hispanic women were expected to agree that canned beans were not true to culture [26]. However, the survey indicated that most disagree with this assertion. The minimal use of canned beans by less acculturated Latinas may be due to reasons other than cultural discrimination alone. Concerns about preservatives, health, price, family acceptance, taste, a general reluctance to use canned or ready-made products, or simply a greater preference for dry beans are alternate explanations. Qualitative research could help discern reasons behind the apparent negative attitude toward canned beans to guide nutrition education approaches [36].

Some women in the current study expressed concern about use of preservatives in canned beans. Park et al. found that Latinas defined healthy foods in terms of purity and freshness, which are qualities typically associated with foods eaten in their countries of origin [37]. Alternatively, Ramirez et al. found that bicultural Hispanic women in California perceived American foods as healthier than some traditional Mexican foods [38]. Lack of access to preferred bean types, forms (dry and canned), and packaging in areas of the US may contribute to reduced bean consumption. Availability of desired ingredients was the most common reason preventing Hispanic women living in Belgium from cooking their traditional cuisine [39]. However, ingredient shortage is unlikely in the American Southwest as compared to Latinas in Iowa or New England [15,19].

For consumers interested in natural, non-genetically modified, or gluten free products, canned or dry beans are minimally processed and are a nutrient-rich, easily accessible, and affordable food that should be recommended as part of a healthy diet [7,10]. A recent survey exploring open-ended definitions of healthy foods found similar desires for less processed, low fat, and high in nutrients between Hispanics and non-Hispanics [40]. While the general public often equates “freshness” with nutrition or health, research shows that similar nutrient profiles are delivered by canned fruits and vegetables, and dry or canned beans [41]. Education about health-related and nutritional similarities between dry and canned beans may be beneficial to dispel misconceptions about canned foods that may inhibit usage and contribute to nutrition inadequacies [42]. Canned foods also last longer than fresh, which can increase food and nutrition security for individuals seeking to economically increase nutritional quality [41].

Although price was an important factor in dry or canned bean selection for all women, Palmer et al. found that designating beans as ‘cheap’ could dissuade rather than encourage low-income women in Iowa from purchase, suggesting an emphasis on value, tradition, or health benefits might be better themes of promotion [42]. Because less acculturated Latinas in this study prefer dry over canned beans and are proficient in preparing dry beans, program materials may want to utilize their skills and focus on supporting this preference. Programming with an emphasis on the equivalent nutrition elements of canned beans with dry beans could encourage Latinas and NHW women to retain beans in their diets with less perceived preparation time [16,43,44]. Education on using dry beans could benefit NHW women who had less knowledge of how to prepare dry beans, but were also concerned with the price of canned. Nutrition education should also address concerns that eating beans will cause intestinal gas or flatulence. Increased flatulence has been suggested to be a transitory effect, or based more on preconceptions. Advice on adding small amounts of beans gradually into the diet could encourage wary consumers to try them until the gut adjusts [45,46].

To counteract acculturation-related decline in bean consumption, culturally sensitive programs should encourage and support retention of traditional healthy eating patterns by low-income Hispanic women. Such programs have been shown to yield better knowledge and health outcomes using healthy adaptations of favorite Mexican American recipes [47]. Emphasis on positive cultural practices may result in more meaningful and attainable health recommendations for immigrants [17,21]. Integration of cultural identities has also led to lower levels of acculturation-related stress and better psychological functioning [48,49]. This study’s findings can be used to appropriately target low-income Latinas based on bean-related purchasing preferences and attitudes in the metro-Phoenix, Arizona region.

This research has several strengths, including addressing the gap in knowledge of dry and canned bean purchasing preferences, bean consumption patterns, and attitudes among low-income Latinas and NHW women in the Southwest. The observed changes in a cultural dietary element like beans help us to better understand the nuances of dietary acculturation among Latinas which appears to be more complex than simply linear [1,4,5]. Additionally, less research has been conducted with female EFNEP participants as compared to women utilizing other food assistance and education programs like WIC, or SNAP. To reduce sampling bias toward bean consumers only, recruitment materials asked about general eating patterns without mentioning beans. Through the collaborative community relationships established with EFNEP and WIC, data collection was facilitated during immigration-related political unrest in Arizona. The insights into the bean views of monolingual Spanish-speaking Latinas who trusted the field staff enough to participate are a unique study strength [50].

Several limitations are to be noted in the study. The data collected were from a convenience sample of mostly Mexican origin Latinas and NHW women who were eligible for government assistance programs. As a non-random sample, these results should not be extrapolated to low-income women from other geographic areas, ethnicities, races, ages, or non-program participants. Since data are cross-sectional, the influence of acculturation processes over time cannot be evaluated. Respondents were not asked if beans were used as a main meal, side dish, or meat substitute, the serving size consumed, nor how beans were prepared. Some of the observed differences between groups by acculturation and ethnicity should be explored through qualitative methods such as interviews or focus groups to better understand the context and meaning of behaviors.

## 5. Conclusions

The results of this study suggest ways to tailor nutrition messages about beans and other pulses based on preferences, attitudes, and purchasing behaviors of low-income Latinas and NHW women in the Southwest US. Further research on food beliefs, preferences, practices, variety and manner of bean use, knowledge of how beans are incorporated into daily diets, and perceptions of canned foods would provide additional insight to aid in the promotion of beans and other pulses to low-income women. A deeper understanding of how acculturation affects bean consumption can direct culturally sensitive programing toward greater use of these beneficial components of traditional Latino diets. Identifying underlying influences on choices by low-income women regarding bean consumption could lead to better-informed programming to meet US DGA recommendations for maintaining overall health and preventing chronic disease.

## Figures and Tables

**Table 1:** Distribution of demographic and household characteristics of low-income Arizona women by Bidimensional Acculturation Scale classifications (*N* = 356).

Characteristics	Total Sample	Less Acculturated Latinas 42% (151)	Bicultural Latinas 25% (88)	More Acculturated Latinas 12% (41)	Non-Hispanic White 21% (76)	*p* Value
	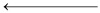 mean ± standard deviation 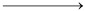					
Age in years (±SD)	33.1 ± 9.2	34.3 ± 7.2	30.6 ± 8.9	27.3 ± 9.3	36.3 ± 10.8	<0.001
Number of children under age 20	2.3 ± 1.3	2.7 ± 1.2	2.4 ± 1.3	2.0 ± 1.3	1.5 ± 1.4	<0.001
Number of adults	2.3 ± 1.0	2.4 ± 1.0	2.5 ± 1.0	2.1 ± 0.8	2.0 ± 0.9	0.011
Total household size	4.6 ± 1.8	5.1 ± 1.6	4.9 ± 1.7	4.2 ± 1.6	3.5 ± 1.8	<0.001
	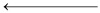 % 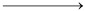					
**Years of Education**						<0.001
6 th grade or less	11.2	25.2_a_	2.3_b_	0_b_	0_b_	
7–9 th grade—Junior High	15.4	27.2_a_	11.4_b_	4.9_b,c_	2.6_c_	
10–11 th grade	14.3	14.6_a,b_	20.5_b_	14.6_a,b_	6.6_a_	
12 th grade or GED	22.8	21.2_a_	25.0_a,b_	39.0_b_	14.5_a_	
Some college—no degree	24.4	6.6_a_	27.3_b_	31.7_b_	52.6_c_	
Associates degree or more	11.8	5.3_a_	13.6_b_	9.8_a,b_	23.7_b_	
**Marital Status**						<0.001
Single/Divorced/Widowed	36.1	17.3_a_	36.4_b_	68.3_c_	55.3_c_	
Married/Cohabitating	63.9	82.7_a_	63.6_b_	31.7_c_	44.7_c_	
**Food Security Status**						0.008
High	48.4	44.8_a_	54.5_a_	48.8_a_	48.0_a_	
Low	36.9	46.9_a_	30.7_b_	24.4_b_	32.0_b_	
Very low	14.7	8.4_a_	14.8_a,b_	26.8_b_	20.0_b_	

Same subscript letters (a, b, etc.) indicate column proportions that are not significantly different from each other.

**Table 2:** Bean purchasing practices and preferences of low-income Arizona women by Bidimensional Acculturation Scale categories (*N* = 356).

Characteristics	Total Sample	Less Acculturated Latinas 42% (151)	Bicultural Latinas 25% (88)	More Acculturated Latinas 12% (41)	Non-Hispanic White 21% (76)	*p* Value
	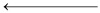 % 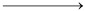					
**How often do you eat beans?**						<0.001
Once a month or less	11.0	4.6_a_	8.0_a,b_	17.1_b,c_	23.7_c_	
2–3 times per month	25.8	13.2_a_	27.3_b_	34.1_b,c_	44.7_c_	
1–2 times per week	29.5	27.8_a_	30.7_a_	39.0 _a_	26.3_a_	
3–4 times per week	22.5	34.1_a_	23.9_a_	4.9_b_	5.3_b_	
5+ times per week	11.2	19.2_a_	10.2_a,b_	4.9_b,c_	0_c_	
**Buys any beans (dry or canned)**	93.3	100 _a_	97.7 _a_	87.8_b_	77.6_b_	<0.001
**Type of bean purchased**						<0.001
Both	44.1	30.5_a_	55.7_b_	63.4_b_	47.4_b_	
Dry bean only	42.7	68.2_a_	37.5_b_	22.0_b,c_	9.2_c_	
Canned bean only	5.9	0.7_a_	4.5_b_	2.4_a,b_	19.7_c_	
Neither dry or canned	7.3	0.7_a_	2.3_a_	12.2_b_	23.7_b_	
**Knows how to prepare dry beans**	90.1	96.7 _a_	95.5 _a_	82.9_b_	75.0_b_	<0.001
**Dry beans cooked in household**						<0.001
Once a month or less	18.6	3.5_a_	9.4_a_	36.6_b_	47.4_b_	
2–3 times per month	28.4	16.8_a_	34.1_b_	29.3_a,b_	43.4_b_	
1–2 times per week	31.6	42.0_a_	38.8_a_	26.8_a_	6.6_b_	
3–4 times per week	14.2	23.1_a_	12.9_a,b_	7.3_b,c_	2.6_c_	
5 or more times per week	7.2	14.7_a_	4.7_b_	0_b_	0_b_	
**Buys dry beans**	86.8	98.7 _a_	93.2_b_	85.4_b_	56.6_c_	<0.001
**For the 87% who buy dry beans:**						
**Preferred dry bean packaging type**						
Loose	42.5	58.8_a_	39.0_b_	28.6_b_	4.7_c_	<0.001
Small bags	44.5	31.1_a_	50.0_b_	48.6_a,b_	76.7_c_	<0.001
Medium bags	23.7	22.3_a_	20.7_a_	42.9_b_	18.6_a_	0.041
Large bags	10.1	13.5	9.8	0	7.0	n.s.
**Important dry bean purchase traits**						
Price	58.9	59.7	52.4	54.3	72.1	n.s.
Tradition—always buy type	36.2	46.3_a_	25.6_b_	37.1_a,b_	20.9_b_	0.002
Quality	26.2	28.9	20.7	28.6	25.6	n.s.
Nutritional value	25.2	26.8	28.0	14.3	23.3	n.s.
Color of beans	24.6	28.9	24.4	20.0	14.0	n.s.
Taste of beans	20.1	18.8	14.6	25.7	30.2	n.s.
Cook quickly	17.2	21.5_a_	11.0_b,c_	5.7_c_	23.3_a,b_	0.037
Brand	14.6	17.4	12.2	11.4	11.6	n.s.
**Prefer beans from a specific country?**						<0.001
No country preference	66.1	58.8_a_	67.9_a,b_	80.0_b_	76.7_b_	
Yes, Mexico or Central America	28.3	41.2_a_	28.4_a_	8.6_b_	0_b_	
Yes, USA	5.5	0_a_	3.7_b_	11.4_b,c_	23.3_c_	
**Buys canned beans**	50.0	31.1_a_	60.2_b_	65.9 _b_	67.1_b_	<0.001
**For the 50% who buy canned beans:**						
**Important canned bean purchase traits**						
Price	48.9	36.2_a_	35.8_a_	40.7_a_	78.4_b_	<0.001
Tradition—always buy type	20.2	21.3	18.9	37.0	11.8	n.s.
Quality	19.1	14.9	13.2	22.2	27.5	n.s.
Nutritional value	18.5	14.9	17.0	18.5	23.5	n.s.
Taste of beans	41.6	34.0	41.5	48.1	45.1	n.s.
Brand	19.7	170	15.1	22.2	25.5	n.s.

Same subscript letters (a, b, etc.) indicate column proportions that are not significantly different from each other; n.s. = not significant.

**Table 3:** Beliefs and attitude scores with respect to general and canned beans among low-income Arizona women by Bidimensional Acculturation Scale category (*N* = 356).

Here Are Reasons People Have Said Prevent Them from Eating or Cooking Beans. Do You Agree or Disagree?	Total Sample	Less Acculturated Latinas 42% (151)	Bicultural Latinas 25% (88)	More Acculturated Latinas 12% (41)	Non-Hispanic White 21% (76)	*p* Value
	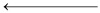 % 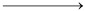					
**1. Difficult to make food with beans**						0.005
Disagree	82.9	84.8_a_	89.8_a_	87.8_a_	68.4_b_	
Neutral	10.6	8.3_a_	4.5_a_	9.8_a,b_	22.4_b_	
Agree	6.6	6.9_a_	5.7_a_	2.4_a_	9.2_a_	
**2. Family/children will not eat**						0.004
Disagree	76.6	73.3_a_	87.5_b_	90.2_b_	63.2_a_	
Neutral	10.7	11.3_a,b_	6.8_b_	4.9_a,b_	17.1_a_	
Agree	12.7	15.3_a_	5.7_c_	4.9_b,c_	19.7_a_	
**3. Do not like taste of beans**						n.s.
Disagree	74.7	70.9	83.0	82.9	68.4	
Neutral	10.7	9.9	8.0	9.8	15.8	
Agree	14.6	19.2	9.1	7.3	15.8	
**4. Only poor people eat beans**						n.s.
Disagree	89.0	87.3	88.6	100.0	86.8	
Neutral	7.3	7.3	6.8	0	11.8	
Agree	3.7	5.3	4.5	0	1.3	
**5. Beans not eaten by friends**						0.013
Disagree	70.5	61.6_a_	78.4_b,c_	87.8_c_	69.7_a,b_	
Neutral	22.8	29.1_a_	18.2_a,b_	12.2_b_	21.1_a,b_	
Agree	6.7	9.3_a_	3.4_a,b_	0_b_	9.2_a_	
**6. Beans cause intestinal gas**						0.003
Disagree	21.4	30.5_a_	19.3_a,b_	15.0_a,b_	9.2_b_	
Neutral	32.1	24.5_a_	30.7_a,b_	45.0_b_	42.1_b_	
Agree	46.5	45.0_a_	50.0_a_	40.0_a_	48.7_a_	
**7. Dry beans take too long to prepare**						<0.001
Disagree	54.4	64.9_a_	52.9_a_	58.5_a_	32.9_b_	
Neutral	17.7	14.6_a_	16.1_a_	22.0_a_	23.7_a_	
Agree	27.9	20.5_a_	31.0_a,b_	19.5_a_	43.4_b_	
**General bean attitude scale** (μ ± SD) (Summary of questions 1–5 †)	20.7 ± 3.7	20.2 ± 3.6	21.7 ± 3.3	22.3 ± 2.6	19.6 ± 4.0	0.001
**8. Canned beans not true to culture**						<0.001
Disagree	74.4	60.7_a_	81.4_b_	97.6_c_	80.3_b_	
Neutral	15.5	21.4_a_	9.3_b,c_	2.4_c_	18.4_a,b_	
Agree	10.1	17.9_a_	9.3_a_	0_b_	1.3_b_	
**9. Family will not eat canned beans**						<0.001
Disagree	56.8	39.1_a_	65.5_b_	77.5_b_	71.1_b_	
Neutral	16.7	18.5_a_	14.9_a_	12.5_a_	17.1_a_	
Agree	26.6	42.4_a_	19.5_b_	10.0_b_	11.8_b_	
**10. If I cannot cook beans myself will go without**						<0.001
Disagree	66.2	60.7_a_	72.4_a,b_	78.0_b_	63.2_a,b_	
Neutral	18.6	14.5_a_	16.1_a_	14.6_a_	31.6_b_	
Agree	15.2	24.8_a_	11.5_b_	7.3_b_	5.3_b_	
**11. Canned beans are not healthy**						<0.001
Disagree	52.2	31.5_a_	55.2_b_	75.6_c_	75.0_c_	
Neutral	32.6	42.0_a_	32.2_a,b_	22.0_b_	21.1_b_	
Agree	15.3	26.6_a_	12.6_b_	2.4_b,c_	3.9_c_	
**12. Canned beans are too expensive**						<0.001
Disagree	58.7	41.8_a_	67.4_b_	68.3_b_	76.3_b_	
Neutral	24.1	32.2_a_	19.8_b_	17.1_a_	17.1_b_	
Agree	17.2	26.0_a_	12.8_b_	14.6_a_	6.6_b_	
**13. Canned beans do not taste good**						<0.001
Disagree	47.8	32.5_a_	52.3_b_	61.0_b_	65.8_b_	
Neutral	19.4	19.2_a_	19.3_a_	19.5_a_	19.7_a_	
Agree	32.9	48.3_a_	28.4_b_	19.5_b_	14.5_c_	
**14. Canned beans have preservatives**						<0.001
Disagree	23.5	16.9_a_	27.1_a,b_	39.0_b_	23.7_a,b_	
Neutral	30.5	19.7_a_	29.4_a,b_	39.0_b,c_	47.4_c_	
Agree	45.9	63.4_a_	43.5_b_	22.0_c_	28.9_b,c_	
**Canned bean attitude scale** (μ ± SD) (Summary of questions 8–13 †)	21.7 ± 4.9	19.2 ± 4.8	22.8 ± 5.0	24.4 ± 3.9	23.8 ± 3.8	<0.001

† Items reverse coded for summation. Same subscript letters (a, b, etc.) indicate column proportions that are not significantly different from each other.
